# The identification of novel single nucleotide polymorphisms to assist in mapping the spread of *Bacillus anthracis* across the Southern Caucasus

**DOI:** 10.1038/s41598-018-29738-3

**Published:** 2018-07-26

**Authors:** Mitat Sahin, Fatih Buyuk, Les Baillie, Roman Wölfel, Adam Kotorashvili, Alexandra Rehn, Markus Antwerpen, Gregor Grass

**Affiliations:** 1 Kafkas University, Kars, Turkey; 2 Cardiff University, Cardiff, Wales UK; 3 Bundeswehr Institute of Microbiology, Munich, Germany; 4 Lugar Center for Public Health Research at the National Center for Disease Control, Tbilisi, Georgia

## Abstract

Anthrax is common as a zoonotic disease in the southern Caucasus area including parts of Turkey and Georgia. In this region, population genetics of the etiological agent *Bacillus anthracis* comprises, where known, the major canonical single nucleotide polymorphism (canSNP) groups A.Br.Aust94 and A.Br.008/009 of the pathogen’s global phylogeny, respectively. Previously, isolates of *B. anthracis* from Turkey have been genotyped predominantly by multi locus variable number of tandem repeat analysis (MLVA) or canSNP typing. While whole genome sequencing is the future gold standard, it is currently still costly. For that reason we were interested in identifying novel SNPs which could assist in further distinguishing closely related isolates using low cost assay platforms. In this study we sequenced the genomes of seven *B. anthracis* strains collected from the Kars province of Eastern Anatolia in Turkey and discovered new SNPs which allowed us to assign these and other geographically related strains to three novel branches of the major A-branch canSNP-group (A.Br.) Aust94. These new branches were named Kafkas-Geo 1–3 and comprised isolates from the Kars region and the neighboring republic of Georgia suggesting a common ancestry. The novel SNPs identified in this study connect the population genetics of *B. anthracis* in the South Caucasus and Turkey and will likely assist efforts to map the spread of the pathogen across this region.

## Introduction

In many parts of the world the zoonotic disease anthrax remains endemic as evidenced by a significant number of human infections. This is particularly the case for Middle Eastern countries including those surrounding the Caucasus mountain ranges. For example the Turkish Ministry of Health reported a total of 26,954 human anthrax cases for the period between 1960 and 2005^1,2^. Turkey tackles this health challenge by employing strict animal vaccination programs, infection reporting systems and outbreak-associated research activities^3–5^. In this country, diverse isolates of *Bacillus anthracis*, the endospore-forming bacterium that causes anthrax, have been sampled from infected humans, animals and from the environment in the past^6–8^. Similarly, in the neighboring country of Georgia anthrax is also endemic^9^ and there is a considerable collection of domestic *B. anthracis* strains^10,11^. Several studies have genotyped Turkish or Georgian isolates by molecular methods providing a preliminary picture of the phylogenetic relationships of *B. anthracis* in this region of Western Asia^6–8,10^. For Georgia there is now a quite detailed map of the genetic diversity of the pathogen present in the country^10^ and for Turkey similar efforts are ongoing^6^. These efforts have been facilitated by applying the original canSNP scheme that has been used to establish a global genetic population structure of *B. anthracis*^12^. In both countries we find *B. anthracis* strains belonging to the canonical SNP (canSNP) groups A.Br.Aust94^8,10^ and A.Br.008/009^10,13^ (also known as the Trans-Eurasian group), respectively. Notably, in Georgia and eastern Turkey the A.Br.Aust94 seems to dominate^8^^,^^10^.

Recently, Turkish and Georgian research groups have teamed up to correlate the phylogeography of *B. anthracis* in Northeastern Turkey and Georgia. Located among this northeastern part of Turkey at the intersection of Anatolia (Asia Minor) and the Caucasus, Kars province is particularly affected by anthrax. While only covering little more than 1% of Turkey, the predominantly rural Kars province suffered 19.7% of all human cases (2,415 in total) of anthrax between 1995 and 2005^1^. From 2009 to 2017 there were an additional 89 human infections^14^ and between 2012 and 2017 a total of 129 animal cases in 73 outbreaks in Kars province reported (Kars Directorate of Provincial Food Agriculture And Livestock, Turkey: https://kars.tarim.gov.tr/; 2017). In their recent study Khmaladze *et al*.^8^ applied 25-(multi)-locus variable-number tandem repeat analysis (MLVA-25) and canSNP-typing in order to genotypically characterize a collection of 30 Turkish and 30 Georgian *B. anthracis* isolates. Similar to earlier findings^10^ all these 60 strains belonged to the single distinct A-branch lineage A.Br.Aust94 which is part of the original canonical SNP-typing scheme for *B. anthracis*^12^ and related isolates were termed A3a in a canSNP predating MLVA-8 typing scheme^15^. This phylogeny was not unexpected because A.Br.Aust94 lineage strains are dominating in the Turkish-Southern Caucasian region and in Georgia^6,11,15^. Recent high resolution genome sequence-based genotyping of Georgian *B. anthracis* strains has provided a reference set of the pathogen’s genetic population structure in the South Caucasus region^10^. The authors also included the analysis of several strains of Turkish origin in their study (but no details) and the results confirmed the close relationship of Georgian and Eastern Anatolian strains within several sublineages of A.Br.Aust94^10^.

While there is now considerable genomic information of the diversity in Georgia^10,13^, insight into the genomic population structure of Turkish anthrax strains must, thus far, mostly be extracted from datasets addressing unrelated topics^12,16^ and include Turkish genomes rather ancillary. In order to obtain a clearer picture on the phylogenetic population structure of Eastern Anatolian *B. anthracis*, we have now analyzed several representative strains from a recent study on isolates from Georgia and the northeastern part of Turkey on a genomic level. This information was used to design and test new SNP-based assays which could be used to further characterize the transboundary spread of the bacterium across the region.

## Material and Methods

### Growth of *B. anthracis* and extraction of DNA from inactivated culture material

Sterile DNA-samples of 30 *B. anthracis* isolates from Kars province (Turkey)^8^ (Table 1) were isolated from overnight cultures on 5% sheep blood agar plates from which several loops of colonies were heat-inactivated by autoclaving at 121 °C for 20 minutes. Sterile genomic DNA was extracted using QIAamp DNA Mini Kits (Qiagen, USA) according to the manufacturer’s instructions. Sterility of DNA-samples was confirmed by culturing of 5% of the final DNA-volume with negative results. Vegetative cells of *B. anthracis* from our strain collection were cultured, inactivated and DNA isolated as described previously^17^. All steps involving live *B. anthracis* were conducted in a biosafety level 3 laboratory. DNA concentrations were quantified using the Qubit dsDNA HS Assay Kit (Thermo Fisher) according to the supplier’s protocol. DNA preparations were stored at −20 °C until further use.

**Table 1:** Metadata of B. anthracis strains belonging to canSNP-group A.Br.Aust94 (clade A.Br.015/013) from Eastern Anatolia (Turkey) used for analysis in this study*.

Strain designation	Alternative name**	Year of isolation	County of sampling	Origin of isolation
Kafkas-2	K-2	<2004	Kars	Cattle
Kafkas-28	K-28	<2004	Kars	Cattle
Kafkas-44	K-86	<2004	Kars	Sheep
Kafkas-51	K-107	2004	Akyaka	Cattle
Kafkas-52	K-62	2004	Kars	Sheep
*Kafkas-60*	*K-68*	2006	Arpacay	Sheep
Kafkas-62	K-78	2005	Kars	Cattle
*Kafkas-68*	*K-132*	2007	Kars	Cattle
*Kafkas-78*	*K-156*	2008	Kars	Cattle
Kafkas-80	K-220	2008	Akyaka	Sheep
*Kafkas-86*	*K-51*	2006	Kars	Cattle
Kafkas-98	K-98	2009	Selim	Cattle
*Kafkas-100*	*K-116*	2009	Digor	Soil
Kafkas-107	K-52	2010	Kars	Cattle
Kafkas-116	K-44	2011	Digor	Cattle
Kafkas-132	K-139	2012	Kars	Cattle
Kafkas-139	K-60	2012	Kars	Sheep
Kafkas-145	K-80	2012	Arpacay	Soil
Kafkas-146	K-183	2012	Kars	Soil
*Kafkas-149*	*K-160*	2012	Selim	Soil
Kafkas-150	K-146	2012	Kars	Soil
Kafkas-156	K-150	2013	Kars	Cattle
Kafkas-160	K-173	2013	Digor	Dog
Kafkas-173	K-204	2013	Kars	Soil
Kafkas-183	K-211	2013	Kars	Human
Kafkas-199	K-149	2013	Selim	Soil
Kafkas-204	K-199	2013	Kars	Soil
Kafkas-211	K-215	2014	Kars	Soil
*Kafkas-215*	*K-100*	2014	Selim	Soil
Kafkas-220	K-145	2014	Kars	Cattle

*Abbreviated information from^8^. **Strain designations according to numbering of column two of Table 2 from^8^ prefixed by “Kafkas” (Kafkas University, Kars). Indicated in italicized and underlined letters are strains with genomes sequenced in this study.

### Whole genome sequencing

For library preparation the Nextera® XT DNA Library Preparation kit (Illumina) was used with an input DNA amount of 1 to 3 ng. Library sequencing was performed on a MiSeq instrument (Illumina) using MiSeq Reagent Kit v3 (600-bp) chemistry (Illumina). High-quality paired-end reads (Q > = 30) were assembled *de novo* using an in-house script based on the SPAdes (version 3.11.1) assembler to create draft genomes^18^. For further improvement of these draft genomes, i.e., correcting SNPs or closing small gaps and INDELs, the genome refining tool Pilon (version 1.22)^19^ was used. These processed scaffolds were manually checked for contaminant reads and uploaded to the NCBI Sequence Read Archive (Bioproject PRJNA421249). Annotation was automatically performed by the NCBI Prokaryotic Genome Annotation Pipeline^20^.

### Analysis of whole genome sequencing data – SNP calling

For rapid core chromosome multiple-alignment, the Parsnp tool from the Harvest Suite was used^21^. For this, representative genomes from public databases (Supplementary Table S1) and newly sequenced strains of *B. anthracis* were aligned against the *B. anthracis ‘Ames ancestor’* reference chromosome (NC_007530) with Parsnp (parameters -c -e -u -C 1000) and called SNPs were extracted into a VCF file using the HarvestTools (version 1.0) from the same software suite. To enhance data quality, closely adjacent SNPs with a distance of less than 10 bp as well as positions harboring undefined nucleotides (“N”) were removed. The “R” analysis package phangorn was used to construct a Maximum-Parsimony-tree based on these high quality SNPs as well as to calculate the consistency index^22^. This edited file was again used as an input file in the HarvestTools to compile a FASTA file comprising the concatenated SNPs as multiple-sequence alignment. SNPs found within the analyzed *B. anthracis* chromosomes can be found in Supplementary Table S2. In addition, a minimum spanning tree was computed in BioNumerics 6.6 (Applied Maths) from the VCF SNP-file (in binary format) as input and manually edited for style.

### Interrogation of SNPs via PCR with high resolution melting curve analysis (HRM-SNP)

In order to validate clade-specific SNPs identified by whole genome SNP-discovery and to determine the distribution of these SNPs in additional *B. anthracis* DNAs, high-resolution melt (HRM) PCR assays were utilized. Primer oligonucleotides were designed surrounding the SNP positions with the Primer-BLAST tool of NCBI^23^ using the *B. anthracis* Ames Ancestor chromosome (accession # NC_007530) as a reference. HRM-SNP primer sequences for real-time PCR-assays are listed in Table 2. Each primer pair was used in a 10 μl single-plex reaction. For this, 0.2 μM of each primer pair, 3 mM MgCl_2_ and approximately 20 ng of template DNA were added to 1 × LightCycler 480 High Resolution Melting Master mix (Roche). Amplification and melting curve analysis was carried out on the LightCycler 480 II (Roche) as described in^17^.

**Table 2:** SNPs and respective primer sequences used for Kafkas-Geo HRM-SNP-assays.

SNP	Position^*^	SNP state	Primer forward/reverse (5′-3′)	Reference
Kafkas-Geo 2	127,121	A/G	CGGCGAGCAAATGTATGAGTTC/ CTTAGAAACACCACGGAAATCACG	This work
Kafkas-Geo 1	4,385,818	T/C	CAATCCGCGCAACCTACAC/ GATCGAGCGCAGTCTTGTG	This work
Kafkas-Geo 3 (A.Br.29)	3,960,657	G/A	GCCGTTCTACATGCAAAGTATTCC/ ATCGTACTTATTGGTGGTACTGC	^10^

^*^In relation to the Ames Ancestor reference genome (NC_007530).

### Data Availability Statement

All data generated or analyzed during this study are included in this published article and its supplementary information files are available in the NCBI Sequence Read Archive (SRA) repository (Accession numbers PPEM00000000- PPES00000000).

## Results

### Selection of isolates for whole genome sequencing and mapping

**Figure 1:**
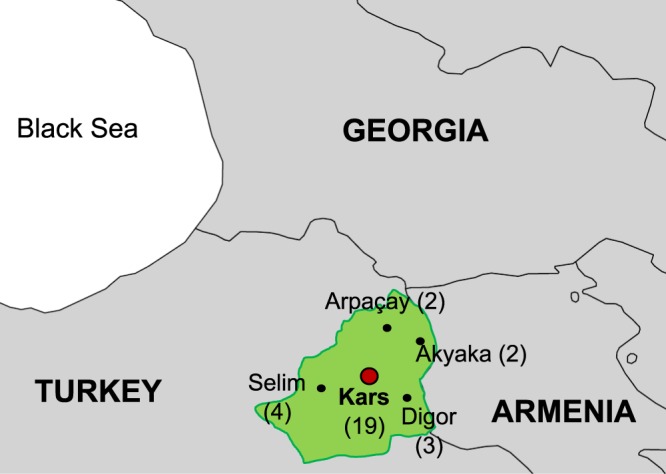
Overview map of the southern Caucasus region. Indicated in green is Kars province with its capital, the city of Kars (red circle). Numbers of strains isolated from diverse Kars province counties (capitals indicated, including Kars city) are given in parenthesis.

Of the 30 strains characterized by canSNP-typing and MLVA-25 from^8^, seven strains (Kafkas-60, -68, -78, -86, -100, -149 and -215) were selected for whole genome sequencing based on their diverse MLVA-profiles, year of isolation and geographical origin (Table 1, Fig. 1). Genome sequencing of these seven “Kafkas” isolates yielded an average number of 703,422 reads (396,027–935,582) per isolate, resulting in an average sequencing depth of >62-fold. De novo assembly produced between 27 and 104 scaffolds (>500 bp) per genome. Each isolate covered at least 95% of the reference chromosome of *B. anthracis str. ‘Ames ancestor’* (NC_007530) and can therefore be assumed to represent most of the core genome.

### Chromosomal SNP analysis suggests a common ancestry of Turkish and Georgian strains of the *B. anthracis* A.Br.Aus94 canSNP lineage

**Figure 2:**
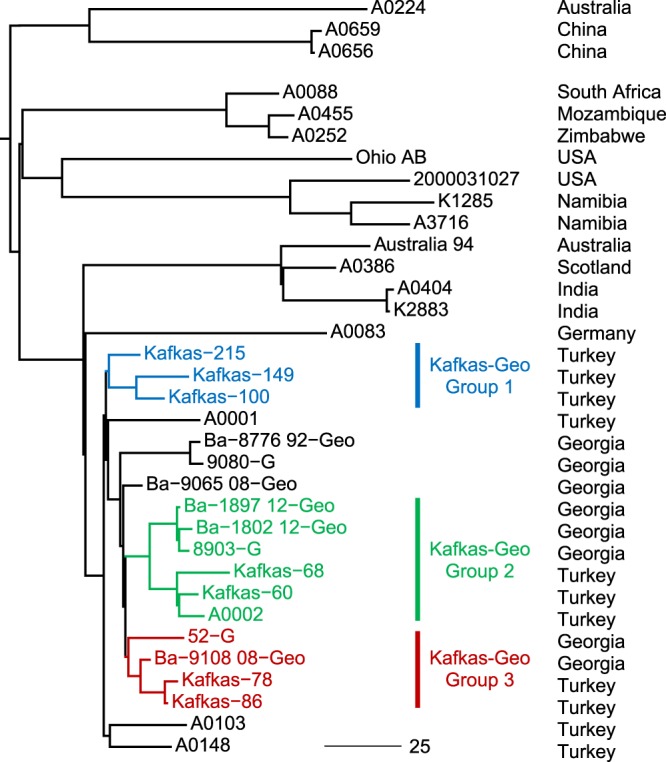
Rooted phylogenetic tree of representative *B. anthracis* strains derived from chromosomal SNPs. A total of 1300 SNPs were used to construct a Maximum-Parsimony tree with a consistency index of 0.999. The right column indicates the country of origin and canSNP group information of representatives (in brackets). The arrow denotes the branch leading to clade A.Br.Aus94 (A.Br.002/014). Genome sequenced strains in this study are drawn in bold letters.

From a chromosomal dataset of the in-house sequenced strains and representatives from public databases (N = 37; Supplementary Table S1) 1300 SNPs were called (Supplementary Table S2) with a consistency index of 0.999. These chromosome-wide SNPs were concatenated and these sequences used to infer the phylogenetic relationships of the analyzed strains with focus on the A.Br.Aust94 lineage (Fig. 2). The chromosome of *B. anthracis* str. ‘Ames ancestor’ was used as reference in order to root the tree. Strains from Turkey and Georgia revealed a common ancestry as they clustered into a single sub-lineage, with a separate sister-group comprising isolates from Germany, Scotland, India and the eponymous Australia94 strain. More distantly related were A.Br.Aust94 strains isolated from Africa, the United States of America, Australia and China (Fig. 2).

### Genome-sequenced *B. anthracis* strains from Eastern Anatolia can be grouped into SNP-related groups comprising additional isolates from Turkey and Georgia

The chromosomal SNP dataset was converted into a binary (0/1) matrix (Supplementary Table S2) as in^16^ and used to draw a minimum spanning UPGMA tree for visualizing the numerical SNP differences between neighboring isolates (Supplementary Fig. S1). This representation, while not being a phylogenetic representation of the data, agreed well with the phylogenetic Maximum Parsimony tree shown in Fig. 2. The A.Br.Aus94 chromosomes formed four smaller sub-clusters with one comprising the Turkish and Georgian strains clearly separated by 6 SNPs from the remainder of A.Br.Aust94 diversity (Supplementary Fig. S1). One of these 6 SNPs comprises SNP A.Br.026 previously identified as leading to the clade of predominantly Georgian strains^10^.

Further SNP discovery revealed a variety of SNPs that could be used to root three defined clades of Turkish and Georgian strains. These clades were named Kafkas-Geo group 1 to 3 (Supplementary Fig. S1). Kafkas-Geo group 1 is defined by a derived allele of SNP 4,385,818 and comprises thus far only three Turkish isolates, all of which genome-sequenced in this study. These genomes were separated from each other by a maximum of only 36 SNPs. Three Turkish and three Georgian strains were grouped to Kafkas-Geo group 2. Their chromosomes differed from each other by a maximum of 39 SNPs. This group is defined by 8 SNPs of which SNP 127,121 was selected as clade-defining SNP. Finally, Kafkas-Geo group 3 is defined by previously discovered SNP A.Br.29^10^ leading to two Turkish and two Georgian genomes (Supplementary Fig. S1).

### High-resolution-melt PCR discriminating assays phylogenetically allocate Southern Caucasus strains of *B. anthracis*

New SNP positions discovered from our genome data analysis (Supplementary Tables S1, S2 and Supplementary Fig. S1) and SNP A.Br.029 from^10^ which were considered useful to separate *B. anthracis* strains into Kafkas-Geo groups 1–3 were developed into high-resolution-melt PCR discriminating assays (Table 2). These assays were then used to interrogate (or confirm) the SNP states of all thirty genomes from Kars province (Table 1). The majority (18 isolates) belonged to Kafkas-Geo group 1 showing the derived allele for the namesake SNP-position (Table 3). Kafkas-Geo group 2 comprised seven isolates and Kafkas-Geo group 3 only those two strains that were genome sequenced. Unexpectedly, three strains (Kafkas-107, -173 and 183) exhibited ancestral alleles for each of the three Kafkas-Geo SNPs. Thus, these isolates did not cluster with any of these groups and therefore branch off between these defined clusters (Table 3). Finally, we also interrogated 19 additional strains from the institute’s strain collection belonging to the A.Br.Aust94 canSNP group for the three Kafkas-Geo 1–3 SNPs. Among these were also Dutch isolates that had been previously analyzed^24^. The Kafkas-Geo SNP groups 1–3 seem to be specific for the Caucasus region as neither of the additionally analyzed strains from different geographic origins belonged to any of Kafkas-Geo SNP groups 1–3. All exhibited ancestral states (data not shown) for all of three defining SNP positions.

**Table 3:** Results of HRM-SNP typing.

SNP (ancestral ANC or derived DER allele)			
Strain	Kafkas-Geo 2 127121 (A/G)	Kafkas-Geo 1 4385818 (A/C)	Kafkas-Geo 3 A.Br.29 (G/A)^*^
Ames Ancestor	ANC	ANC	ANC
Kafkas-2, *60*, *68*, 98, 199, 204, 211	DER	ANC	ANC
Kafkas-28, 44, 51, 52, 62, 80, *100*, 116, 139, 132, 145, 146, *149*, 150, 156, 160, *215*, 220	ANC	DER	ANC
Kafkas-*78, 86*	ANC	ANC	DER
Kafkas-107, 173, 183	ANC	ANC	ANC

^*^This and A.Br. 26 to 33^10^. Indicated in italicized and underlined letters are strains with genomes sequenced in this study.

## Discussion

Within the original canSNP typing scheme for *B. anthracis*^12^, canonical lineage A.Br.Aust94 is defined by the allelic state of two canSNPs, A.Br.2 (ancestral allele) and A.Br.3 (derived allele), thus constituting canSNP group A.Br.002/003. Recently, this typing scheme has been amended^16^ in order to acknowledge the pathogen’s increasing genetic diversity discovered within the previous decade. According to the new scheme terminal lineage A.Br.Aust94 is now defined by a derived allelic state of new canSNP A.Br.014^25^. This clade, A.Br.Aust94 (now defined as A.Br.002[ancestral]/A.Br.014[derived]), is geographically vastly distributed as members have been isolated from Australia, Africa (South Africa, Namibia, Mozambique, etc.), Asia (Turkey, Georgia, Thailand, India, China, etc.), America (USA) and Europe (Germany, Great Britain, The Netherlands, etc.)^10,16^. Genotyping confirmed the close relationship of Georgian and Eastern Anatolian strains with isolates of both countries falling into new derived sub-lineages of the A. Br.015/013 node within canSNP group A.Br.Aust94^25^. Further typing with “GeoSNPs”^10^ of the Eastern Anatolian strains (Table 1) revealed that these strains clustered along the lineages A.Br.027/026, A.Br.029/028, or A.Br.030/029, respectively^8^.

In the work at hand we have built upon the previous SNP-typing schemes^10,25^ and added three new defined groups, Kafkas-Geo 1–3 comprising newly sequenced genomes from Kars province as well as previously genotyped genomes from Turkey and Georgia. Combining new genome-based and earlier PCR-derived information^8^ we were able to group all thirty strains from Kars province into the improved typing scheme (Supplementary Fig. S1, Table 3). However, one has to be mindful that there still is a discovery bias since the strains that are in collections are certainly not all representative but just random snap-shots of the diversity present in the southern Caucasus region. Furthermore, not all strains are accessible for genome sequencing yet, making phylogenetic evaluation a bit fuzzy when relying only on MLVA-analysis and partial SNP interrogation.

This has also been a challenge for the analysis of the “Kafkas” strains. Three of the thirty strains from Kars province (Kafkas-107, -173 and -183) exhibited ancestral SNP states for all three Kafkas-Geo SNP-groups (Table 3). Further SNP-based genotyping using published SNP information^10,25^ confirmed the strains’ placements between Kafkas-Geo SNP-groups 1–3 as published for SNPs A.Br. 26–33 in^8^. Notably, strains Kafkas-107 and -183 which are defined as A.Br.029/028 and strain Kafkas-173 as A.Br.028/027, respectively^8^, did not cluster with Kafkas-Geo SNP-groups 1–3. In a previous MLVA-derived tree^8^ Kafkas-107, -173 and -183 are wedged between the clade of strains that are now placed within Kafkas-Geo group 2 together with the two strains (Kafkas-78 and Kafkas-86) now known to belong to Kafkas-Geo group 3. In our new SNP-based scheme (Supplementary Fig. S1) strains Kafkas-107 and -183 would branch off between positions “d” (SNP A.Br.028) and f (A.Br.29) possible sharing derived SNPs with strain Ba-9065/08-Geo. Conversely, strain Kafkas-173 would branch off earlier, between one of the SNPs labeled “b” (A.Br.026) and the four SNPs “c” comprising A.Br.027 (SNPs A.Br. 026–29,^10^). From this theoretical node between “b” and “c” other strains from Turkey (A001, A0103 and A0148) also radiate out. At this point it is not known with which of these strains isolate Kafkas-173 shares any derived SNP states, though. Additionally, Dutch strain CVI-188678-1 (A.Br.027/026)^24^ is possibly very closely related to Kafkas-173 and other Turkish strains from Supplementary Fig. S1 as these and Turkish isolates A001, A0103 and A0148 were defined by identical SNP-states (A.Br.027/026).

Using the SNP-based information related to A.Br. 026–033 from^10^ on 30 strains from Georgia^8^ it is possible to allocate these isolates to the clades depicted in Supplementary Fig. S1. Georgian strains (all strains originally suffixed “–G”) 1242, 6150, 6671, 8295 belong to Kafkas-Geo group 3 (derived for SNP A.Br.029) and strains 89, 91, 154, 406, 762, 1998, 7763, 8263, 8276, 8889, 8903, 9105, 9107, 9450 (A.Br.029/028) could belong to Kafkas-Geo group 2 or to a lineage including Georgian strain Ba-9065/08-G^13^. Less certain is the placement of strains 368, 392, 411, 8347, 8500, 8670 and 9630 (all A.Br.028/027) branching off prior to position “d” (SNP A.Br.028) but after any position labeled “c” (A.Br.027) in Supplementary Fig. S1. Thus, these isolates may be most closely related to genome sequenced strains 9080-G^10^ and Ba-8776/92^13^. Finally, single isolate 52-G which was previously located at a terminal branch because it was found to exhibit derived allelic states for all SNPs tested^10^, constitutes a sub-branch within Kafkas-Geo-group 3 (all members derived for A.Br.029) in the current model (Supplementary Fig. S1). To this group, thus also belong Georgian strains from^8^ named 50, 9099, 9102 and 9104.

In conclusion, from this and a wealth of previous work it has become clear that the A.Br.Aust94 canonical lineage is very well ecologically established in Georgia, (Eastern) Turkey^6,8,10,11^ and possibly other countries of the southern Caucasus region. Recent genome sequencing efforts of further strains isolated from this geographical region and SNP discovery make it now possible to draw an ever increasing resolution map of the genetic diversity of *B. anthracis*. It will be interesting to see to which degree the A.Br.Aust94 lineage with its respective clades reaches into the neighboring countries. These phylogeographical genome typing efforts will also help us to better understand how the European, American or Australian strains which were likely imported from regions where A.Br.Aust94 lineage is ecologically established^10^, fit into the picture. On a smaller scale, in case of Turkey, it has been suggested that (infected) livestock transport between East Anatolia and Western Turkey was the primary route of *B. anthracis* distribution and thus likely importation of A.Br.Aust94 strains into the Western part of the country^6^. The dominance of the A.Br.Aust94 lineage emphasizes the uniqueness of the Kars region compared to the rest of Turkey and highlights the strong geographic structuring. This is similar to what is found in Georgia and suggests effective management to restrict pathogen dispersal.

## Electronic supplementary material

Tables S1+S2

Fig. S1
