# Supplementary material for: The identification of novel single nucleotide polymorphisms to assist in mapping the spread of Bacillus anthracis across the Southern Caucasus
Source: Sci Rep. 2018 Jul 26;8:11254. doi: 10.1038/s41598-018-29738-3 (PMC6062627; doi:10.1038/s41598-018-29738-3)
Supplement: Supplementary file 2 — Fig. S1 [file 41598_2018_29738_MOESM2_ESM.docx]

**The identification of novel single nucleotide polymorphisms to assist in mapping the spread of *Bacillus anthracis* across the Southern Caucasus**

Mitat Sahin, Fatih Buyuk, Les Baillie, Roman Wölfel, Adam Kotorashvili, Alexandra Rehn, Markus Antwerpen, and Gregor Grass

**Supplementary Fig. S1 online: Position of seven Anatolian (“Kafkas”) *B. anthracis* strains within three new lineages (Kafkas-Geo groups 1-3) on a minimum spanning tree based on chromosomal SNPs.** A minimum spanning tree was inferred from 1300 chromosomal SNPs. Numbers next to branch-lines indicate SNPs separating nodes or strains. (A) Shown in the overview are representative genomes of neighboring branches A.Br.Ames, A.Br.001/002 and A.Br.003/004 and the branch (arrow) leading to clade A.Br.Aus94 (A.Br.002/014). Three groups (Kafkas-Geo 1-3) of closely related South Caucasus (“Kafkas”) Anatolian and Georgian strains are indicated in color. (B) Close-up of the Kafkas-Geo groups among their closest relatives. Closed circles indicate Turkish, open circles Georgian isolates. Filled boxes with lower case letters (and thick branch lines) indicate positions of previously identified [^10^](#_ENREF_10) and new SNP positions (numbers indicating their positions in the Ames ‘Ancestor’ genome); a: A. Br.13 –3,101,332, b: A. Br.26 – 3,640,599, c: A. Br.27 – 4,355,524, d: A.Br.28 – 791,256, e: A.Br.32 – 3,520,170+A.Br.30 – 3,528,668+A.Br.33 – 3,610,151). SNPs defining Kafkas-Geo groups 1 to 3 comprise h: KafGeo1 – 4,385,818 at the base of Kafkas-Geo group 1, g: KafGeo2 - 127,121 (one of eight SNPs) leading to Kafkas-Geo group 2 and f: A. Br.29 – 3,960,657 [^10^](#_ENREF_10) separating Kafkas-Geo group 3.
